# Localization and surgical approach to mediastinal parathyroid glands

**DOI:** 10.1186/s13019-022-02052-w

**Published:** 2022-12-07

**Authors:** Ian A. Makey, Laura E. Geldmaker, John D. Casler, Magdy M. El-Sayed Ahmed, Samuel Jacob, Mathew Thomas

**Affiliations:** 1 Department of Cardiothoracic Surgery, Mayo Clinic, 4500 San Pablo Rd, Jacksonville, FL 32224 USA; 2 Department of Otorhinolaryngology/Head and Neck Surgery, Mayo Clinic, Jacksonville, FL USA; 3 Graduate Research Education Program, Mayo Clinic Graduate School of Biomedical Sciences, Mayo Clinic College of Medicine and Science, Jacksonville, FL USA

**Keywords:** Ectopic, Hyperparathyroid, Parathyroidectomy, Sestamibi, SPECT, 4-Dimensional CT, Thymectomy

## Abstract

**Background:**

Hyperactive parathyroid glands (PTGs) are in the mediastinum 4.3% of the time. Historically, localization and resection of these glands can be challenging.

**Methods:**

We searched all operative notes involving a thoracic surgeon and a preoperative diagnosis of hyperparathyroidism from 2001 to 2019.

**Results:**

Eighty-five cases were reviewed, of which 63 were included. Only 14 patients (22%) had de novo hyperparathyroid operations. Seventeen patients (27%) had single-photon emission computed tomography with computed tomography fusion (SPECT-CT) as the only preoperative localization test (excluding chest radiography and ultrasound), and all were resected successfully. The initial surgical approach was transcervical for 16 (27%) patients, however only 7 remained transcervical. 4 (6%) patients had an exploration in which the target lesion was resected but it was not parathyroid tissue.

**Conclusion:**

Most patients presenting with mediastinal PTG have had prior HPT surgery. The trend toward more focused HPT surgery may mean more de novo mediastinal PTG resections. An unambiguous functional and anatomic localization test, such as a spect-ct scan, is the best predictor of a successful resection. Ambiguous or discordant scans should be approached cautiously, and additional confirmatory tests are recommended. For suspected PTG located in the thymus, the thoracic surgeon should choose the most familiar approach to achieve complete thymectomy.

## Introduction

Ectopic parathyroid gland is one of the reasons why surgery for hyperparathyroidism (HPT) is not successful 2–4% of the time. Ectopic glands located in the upper mediastinum can be reached by endocrine surgeons via a cervical incision, however some glands are located deeper in the chest, such as in the thymus gland, and require a thoracic approach. We aim to find the optimal localization studies and surgical approach for mediastinal PTG. We aim to reduce the number of negative explorations for mediastinal PTGs.

According to a meta-analysis, PTGs are located in the thyrothymic ligament 17% of the time and in the mediastinum 4.3% of the time [1]. In patients with HPT, the incidence of mediastinal PTGs increased to 5.2% [1]. Most thyrothymic and many mediastinal glands can still be resected via a cervical exploration [2, 3]. Mediastinal exploration, such as video-assisted thoracoscopy (VATS) or sternotomy, is needed in only 1.5–2% of cases of HPT [4–6] If a PTG cannot be found after a thorough cervical exploration, experts recommend terminating the procedure and bringing the patient back for reimaging [7]. The initial surgeon should not proceed with a sternotomy and continue hunting for the missing gland as there has been a trend away from sternotomy and toward minimally invasive mediastinal exploration [7]. Additional localization studies are recommended because the mediastinum contains a lot of fat and thymic tissue that can hide the PTG [2, 7–9].

## Methods

We obtained Institutional Review Board approval (Application #19-005711) to search operative notes between 2001 and 2019 from 3 Mayo Clinic locations. Search criteria included both a preoperative diagnosis of HPT and the presence of a thoracic surgeon. Patients were excluded if the thoracic surgeon did not participate in the parathyroid surgery.

A transcervical approach was defined as an attempt at resection of the mediastinal PTG through a standard suprasternal horizontal incision. Transcervical approach did not include mediastinoscopy or partial sternotomy, which were categorized separately. Transthoracic approaches included VATS, robotic-assisted thoracoscopy, sternotomy, thoracotomy, and Chamberlain approach. Surgical approach was categorized based on the final approach used.

PTG locations were determined from the operative notes rather than the imaging. Surgical success was defined as removal of PTG tissue. Imaging sensitivity and specificity were calculated with the assumption that the surgical pathology was the criterion standard regarding the presence or absence of PTG tissue. Patients with multiple imaging scans were categorized according to the result of the most recent scan.

## Results

**Fig. 1:**
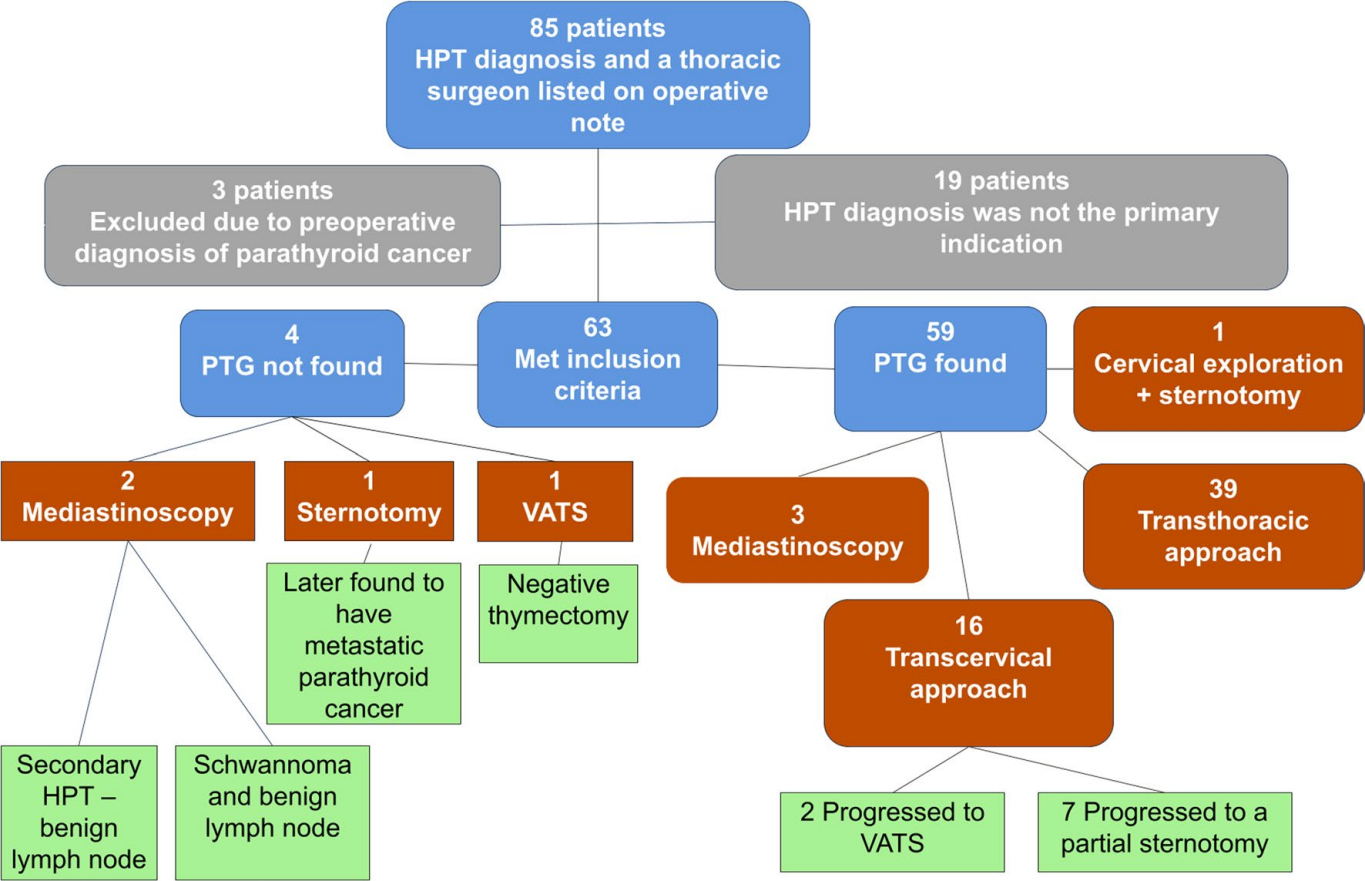
Flow chart of patients from initial identification criteria to final outcome. Flow was divided according to the success of finding parathyroid tissue. Green boxes represent those cases when a PTG was not found or there was a change in surgical approach. Transthoracic approaches included VATS, robotic-assisted thoracoscopy, sternotomy, thoracotomy, and Chamberlain approach

Eighty-five patients met the search criteria. Nineteen patients were excluded because HPT was a diagnosis but not the primary diagnosis. Three more were excluded because they had a preoperative diagnosis of parathyroid cancer, leaving 63 patients (Fig. 1). Two patients were incidentally found to have parathyroid cancer and were included in the analysis. Forty-nine (78%) patients had prior cervical explorations for HPT, some multiple times (Table 1). 3 (5%) patients had prior chest surgery for HPT.

**Table 1:** Demographics

Characteristic	Mean ± SD or percent	N
Age (years)	53 ± 15	63
BMI (kg/m)	30 ± 8	63
Sex		
Male	37%	23
Female	64%	40
Race		
White	97%	61
Other	3%	2
Operative indication		
Primary Hyperparathyroidism	97%	61
Secondary Hyperparathyroidism	2%	1
Tertiary Hyperparathyroidism	2%	1
Preoperative PTH level (pg/mL)(15–65 is normal)	182 ± 185	61
# of prior cervical explorations		
0	22%	14
1	43%	27
2	25%	16
3	10%	6

A variety of imaging techniques were used to localize the hyperactive PTGs (Table 2). Every patient had a sestamibi nuclear medicine scan, which consisted of at least 1 of the following: planar sestamibi, SPECT, or SPECT-CT. SPECT-CT was the most common imaging study. The 4 patients who did not have a SPECT-CT had SPECT and a dedicated CT scan with contrast. SPECT-CT had the highest sensitivity and specificity rate.

**Table 2:** Frequency, sensitivity, and specificity of preoperative localization tests (N = 63)

Study	No. (%)	Positive result	True positive	False positive	Negative result	True negative	False negative	Sensitivity (%)	Specificity (%)
Sestamibi^a^	63 (100)	55	54	1	8	3	5	90	75
SPECT-CT	58 (92)	53	52	1	6	3	3	93	75
4D-CT	29 (46)	24	23	1	5	2	3	86	67
MRI	9 (14)	6	5	1	3	1	2	67	50
Venous sampling	8 (13)	6	5	1	2	1	1	75	50

^a^Sestamibi includes sestamibi planar, SPECT, and SPECT-CT

**Fig. 2:**
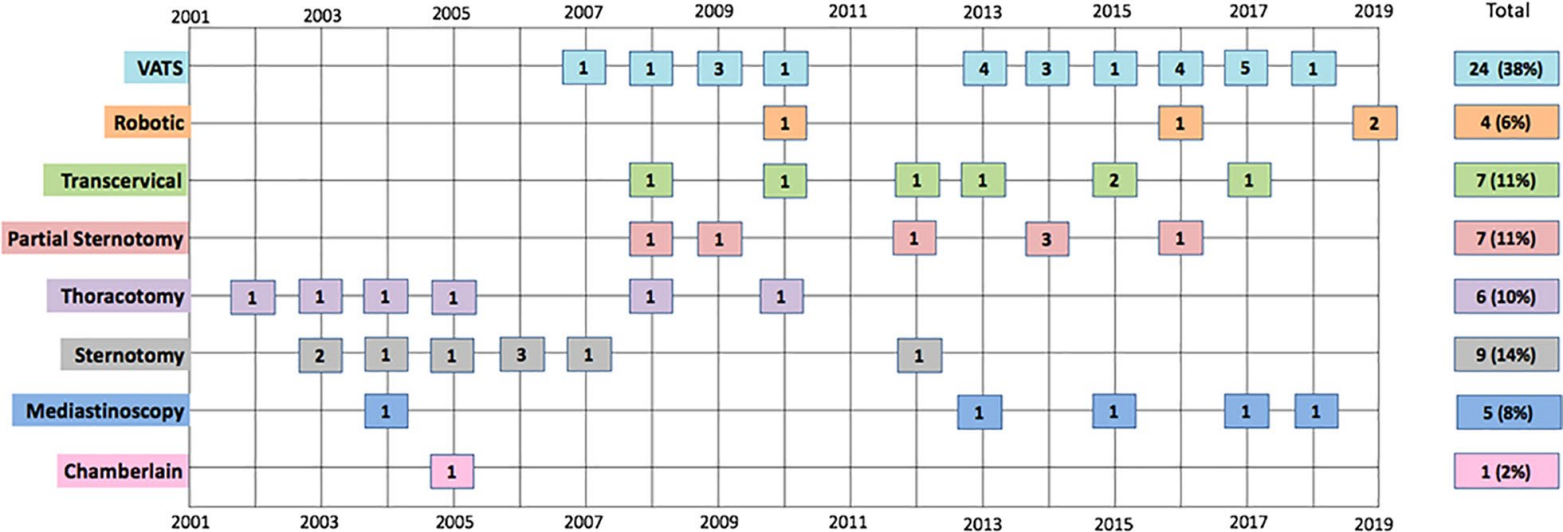
Timeline of 63 procedures reviewed by final operative approach. The number of surgeries performed each year is indicated with a number in the respective box. Those involving a conversion are classified by the final approach performed

Of the 59 successful surgeries, 39 (66%) were transthoracic (Fig. 1). There were 16 (25%) transcervical approaches. An endocrine surgeon initiated the transcervical exploration, and the final approach was dictated by the thoracic surgeon. Of the 16 transcervical approaches, only 7 remained transcervical. 7 progressed to a partial sternotomy and 2 progressed to a VATS approach. Minimally invasive approaches became more common over the course of this 18-year review (Fig. 2). Prior to 2010, sternotomy was the approach used most often, but after 2010, VATS became the most common approach.

**Fig. 3:**
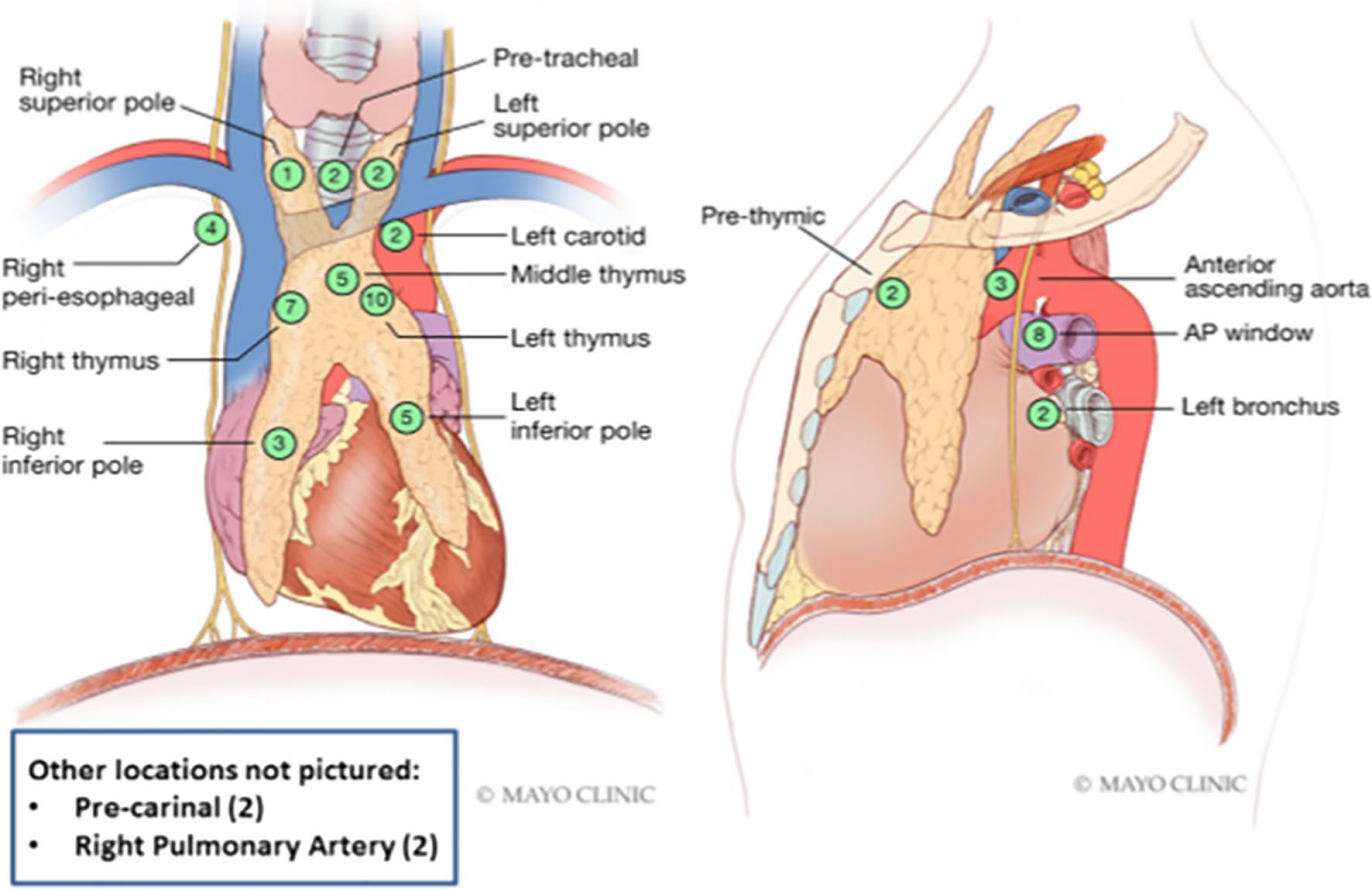
Parathyroid gland operative locations. Illustration of the various operative locations where the parathyroid glands were found throughout the mediastinum. The numbers in the green boxes represent the number of PTG found at each location. Fifty-nine patients had successful parathyroidectomies. One patient had 2 ectopic parathyroid glands excised; therefore, a total of 60 glands are represented in this image. AP, aortopulmonary

The PTGs were located throughout the mediastinal cavity (Fig. 3). One patient had 2 ectopic glands, resulting in a total of 60 PTGs found. 33 PTGs (55%) were found in the thymus. The next most common location was the aortopulmonary window (8 [13%]). We had a 94% success rate for locating mediastinal PTGs. 4 patients had operations in which the PTG was not found. In these cases, the target lesion was resected, but it was not PTG tissue.

## Discussion

How often a hyperactive PTG is in the mediastinum varies according to the definition; some authors define it anatomically, while others define it according to the approach taken. For example, Hu et al. [9] defined a mediastinal PTG as being located below the level of the clavicle. By contrast, Wang et al. [10] described it as inaccessible from the cervical approach and thus requiring (in the 1980s) a median sternotomy. Most authors equate intrathymic PTGs with mediastinal [11], while others include glands adjacent to the thymus [12, 13] Due to these varying definitions, reported incidence rates range from 1.5–25% [14], with a meta-analysis average of 4.3% [1]. Some studies included in the meta-analysis were cadaver studies. Analyzing only those studies in which patients had HPT, the rate of mediastinal PTGs increased to 5.2% [1].

How many mediastinal PTGs require transthoracic exploration? A majority of PTGs located below the clavicle can be resected through a transcervical approach. In a series from 1981 to 2,770 PTG explorations for primary HPT, only 59 (2.1%) required a sternotomy [5]. Randone et al. [4] reported 1.5% of patients with HPT in his institution required transthoracic exploration. Wei et al. [6] reported 1.9% of their patients with HPT were treated with VATS and another 0.3% with mediastinoscopy. This suggests that of the 5% of hyperactive mediastinal PTGs, more than half can be excised via a transcervical approach, typically by the endocrine surgeon.

It should be noted that mediastinoscopy was categorized separately from transcervical exploration. Although mediastinoscopy is technically a transcervical approach, it refers to the anatomic plane anterior to the trachea and posterior to the great vessels and is not typically performed by endocrine surgeons. On the other hand, a transcervical exploration for HPT typically means exploration of the superior thymus gland, which lies anterior to the brachiocephalic artery and vein. Endocrine surgeons may have more familiarity with this anatomy because ectopic thyroid tissue is often located in this area. Since they connote different anatomic areas and require different surgical techniques, we felt distinguishing them would be useful. In this series, mediastinoscopy was used in 5 patients. All of the target lesions were located anterior to the trachea or carina. 2 of those operations did not yield parathyroid tissue (Fig. 3 only shows successful PTG resection). Mediastinoscopy is an excellent minimally invasive approach to the pretracheal space, however it may have a limited role in ectopic mediastinal PTGs since only 3 (5%) out of 60 PTGs were found in the pretracheal space.

The 63 patients described here had a thoracic surgeon perform a mediastinal exploration. When the thoracic surgeon performed a transcervical exploration, a substantial portion of thymus gland was resected, typically down to or past the innominate vein. In our series, 16 resections (27.1%) were attempted by a transcervical approach, all of which were initiated by an endocrine surgeon, with a thoracic surgeon continuing with the deeper transcervical exploration. Only 7 were successfully completed via that approach. If the lesion could not be found, then the thoracic surgeon had to decide what approach to take. In 7 patients, a partial sternotomy was performed, and in 2 cases, the patient was repositioned and a VATS thymectomy was performed. Although the transcervical approach was not optimal in this series, this was a selective series in which thoracic surgeons were involved. It does not include the larger number of operations in which mediastinal PTG tissue was obtained by endocrine surgeons via a transcervical approach. For the thoracic surgeon, it can be a challenge to decide when to attempt a transcervical versus a transthoracic approach, especially once a cervical incision has already been made. Iihara et al. [2] suggest a transcervical approach for those lesions localized preoperatively at the aortic arch or higher and a transthoracic approach for those lesions located lower. In addition to lesion location, the approach taken depends on patient preference and surgeon experience with transcervical versus transthoracic thymectomy. In agreement with other authors [4, 6, 15–18], we prefer a robotic transthoracic approach for any lesion located within the thymus gland. A transthoracic approach permits total resection of mediastinal fat and thymus, so that the culprit lesion is removed en bloc, without capsular rupture [18]. This also adequately treats those rare cases when there is more than 1 PTG located in the thymus gland or the lesion is parathyroid carcinoma [10, 19, 20].

Historically, the rate of negative mediastinal explorations in the literature is substantial and much higher than for cervical or transcervical explorations. In the 1980s, Russell et al. [5], Wang et al. [10], and Conn et al. [13] reported negative exploration rates of 36% (21/59), 36% (17/47), and 29% (6/21) after sternotomy, respectively. With better localization technology, negative exploration rates have decreased. In 1992, Doherty et al. [8] reported a negative exploration rate of 0% for 24 patients receiving sternotomy after unsuccessful angiogram ablation. In the 2000s, transthoracic mediastinal exploration moved to minimally invasive approaches. In 2010 and 2011, Randone et al. [4] and Wei et al. [6] reported negative exploration rates of 23% (3/13) and 12% (2/17), respectively, for VATS exploration. Within the last few years, smaller series (n = 5 and n = 8) with robotic approaches have reported 0% negative exploration rates [17, 18]. We report a negative exploration rate of 6% with a combination of approaches for all forms of HPT including an undiagnosed metastatic parathyroid cancer. Here, we describe the 4 negative explorations in more detail.

The first patient had secondary HPT with PTH levels greater than 3,000, despite maximal medical treatment. He had 2 prior cervical explorations with all 4 glands removed and a portion reimplanted in the forearm. PTH levels remained above 900. Further work-up with SPECT-CT showed a focus of radiotracer activity within the mediastinum with no definite correlation on low-dose CT. A 4D-CT scan revealed a well-defined 1-cm lesion with arterial enhancement anterior to the tracheal bifurcation which corresponded with the SPECT-CT. A mediastinoscopy was performed, revealing a nodule with a vascular pedicle; however, this turned out to be only reactive lymphoid tissue. This was the only false positive SPECT-CT. The other 3 patients’ most recent SPECT-CT scans were negative.

The second patient had 3 PTGs removed and a hemithyroidectomy during a previous cervical exploration. The fourth gland was not found, and the patient’s HPT persisted. Repeat SPECT and ultrasound were negative. MRI revealed 2 enhancing lesions, 1 in the left carotid space and the other anterior to the trachea and posterior to the aorta. Upon exploration, the carotid lesion was a schwannoma. Mediastinoscopy of the paratracheal lesion revealed only benign lymph nodes. It was felt further exploration would not yield additional information. A follow-up 4D-CT was negative for any additional suspicious lesions.

The third patient had 4 previous neck explorations, with 4 PTGs removed, 3 on the right side and 1 on the left. The whole thyroid gland was also resected. An earlier SPECT scan showed a very small area of increased uptake in the cervical thymus to the left of midline, but a more recent SPECT did not show enhancement. The patient also had a neck ultrasound, positron emission tomography, venous sampling, and MRI of the chest and abdomen, all of which were negative. Parathyromatosis was considered. As the patient was still symptomatic and not tolerating medical therapy, a total thymectomy via a sternotomy was performed. No PTGs were found and the patient’s HPT remained. Two months later, the patient had lung nodules removed which were consistent with metastatic parathyroid disease. This patient was not excluded from our analysis because the diagnosis of metastatic parathyroid cancer was not made until 2 months after the index operation.

The fourth patient had a previous bilateral cervical exploration with the left inferior PTG removed; however, their HPT continued. SPECT and 4D-CT were negative but venous sampling showed a PTH level of greater than 3,000 draining from the thymic vein. The patient had a right VATS with total thymectomy. Pathology showed benign thymic tissue without a PTG. The patient’s PTH remained elevated at 161 pg/mL and treatment with cinacalcet continued.

In summary, the false-positive localization scans included a SPECT-CT, an MRI, contradictory SPECT-CTs, and venous sampling. Similar to cervical exploration, certain factors increase the likelihood of negative mediastinal exploration, including a diagnosis other than primary HPT, redo surgery, and ambiguous or discordant localization tests. We routinely send target lesion tissue for frozen section testing to confirm parathyroid tissue.

### Redo surgery

Unfortunately, redo surgery for mediastinal PTG is more the rule than the exception. The rate of redo surgery for mediastinal PTG ranged from 25 to 60% in 3 prior series [6, 17, 18] and 78% in our series. The field of parathyroid surgery has moved to more focused exploration over the past few years [21], and we saw this trend for mediastinal PTGs. Of those 14 patients with no prior HPT operations, half were treated in the last 4 years versus the prior 14 years. This suggests that early referral to a thoracic surgeon for mediastinal or intrathymic PTGs may preclude the need for cervical exploration. That said, redo surgery for a mediastinal PTG may not be as difficult as for a cervical PTG. Depending on the location and prior surgery, the mediastinal PTG may not be in the prior operative field. Multiple prior operations, however, should be approached cautiously out of concern for ambiguous localization studies or misdiagnosis.

### Ambiguous or discordant localization tests

Treatment of patients with ambiguous or discordant localization tests is challenging [4, 6]. All of our unsuccessful cases had discordant or ambiguous localization studies. Localization algorithms for redo cervical HPT surgery recommend 2 concordant noninvasive imaging tests [22]. If the results are discordant or inconclusive, then additional testing is warranted and may include invasive procedures such as selective venous sampling or needle biopsy [23]. We agree with this approach. SPECT-CT is a sensitive test, but in 3 instances it gave a false negative result. The following 3 patients had negative SPECT scans and parathyroid tissue was found.

The first patient had a previous 3.5 PTG resection, but their HPT persisted. Subsequent ultrasound and SPECT-CT were negative. A 4D-CT scan demonstrated a 5-mm avidly enhancing nodule in the thymus with near complete washout on venous phase. A transcervical exploration was followed by a VATS right thymectomy, which contained the PTG.

The second patient had a prior bilateral cervical exploration with a single gland removed, but their HPT remained. Further work-up showed a negative SPECT-CT, a negative MRI with contrast, and a negative arteriogram. Venous sampling was positive for a source within the mediastinum. The patient underwent a total thymectomy via a sternotomy. Intraoperative PTH dropped and only pathologic examination revealed PTG adenoma within the thymus gland.

The third patient had 2 previous cervical explorations with removal of the thyroid gland and 2 PTGs, but their HPT continued. Further work-up revealed 2 negative ultrasounds, a negative MRI, and 2 negative SPECT-CT. Venous sampling was positive from the thymic veins. The patient underwent a cervical exploration with removal of 2 normal appearing PTGs without a drop in PTH. Next, a sternotomy and total thymectomy were performed. A 1-cm mass was found in the left portion of the thymus gland, which was identified as PTG tissue on pathologic examination.

In summary, a CT scan with contrast and 2 venous sampling tests helped convince the surgeons to perform a total thymectomy despite a negative SPECT. This highlights the importance of additional localization tests if the SPECT-CT is ambiguous or negative. On the other hand, there may be no need for redundant localization tests if there is an unambiguous SPECT-CT scan. Since SPECT-CT combines functional (sestamibi) and anatomic (CT) features, it can function well as a stand-alone test. For example, 27% of patients in our series had SPECT-CT only (not including cervical ultrasound or chest radiography), and all were resected successfully.

## Conclusion

Most patients presenting with mediastinal PTG have had prior HPT surgery. The trend toward more focused HPT surgery may mean more de novo mediastinal PTG resections. An unambiguous functional and anatomic localization test, such as a spect-ct scan, is the best predictor of a successful resection. Ambiguous or discordant scans should be approached cautiously, and additional confirmatory tests are recommended. For suspected PTG located in the thymus, the thoracic surgeon should choose the most familiar approach to achieve complete thymectomy.

## Data Availability

The datasets used and/or analysed during the current study are available from the corresponding author on reasonable request.

## Abbreviations

CT: Computerized tomography
HPT: Hyperparathyroid
MRI: Magnetic resonance imaging
PTG: Parathyroid gland
SPECT: Single photon emission computerized tomography
SPECT-CT: Single photon emission computerized tomography combined with a traditional CT scan
SD: Standard deviation
VATS: Video assisted thorascopic surgery
4-D CT: 4-Dimensional CT that visualizes the uptake and washout of contrast by the parathyroid glands
